# Secondary White Matter Injury and Therapeutic Targets After Subarachnoid Hemorrhage

**DOI:** 10.3389/fneur.2021.659740

**Published:** 2021-07-15

**Authors:** Xufang Ru, Ling Gao, Jiru Zhou, Qiang Li, Shilun Zuo, Yujie Chen, Zhi Liu, Hua Feng

**Affiliations:** ^1^State Key Laboratory of Trauma, Burn and Combined Injury, Department of Neurosurgery, Southwest Hospital, Third Military Medical University (Army Medical University), Chongqing, China; ^2^Chongqing Key Laboratory of Precision Neuromedicine and Neuroregenaration, Southwest Hospital, Third Military Medical University (Army Medical University), Chongqing, China; ^3^Department of General Practice, Audio-Visual Education Center, Third Military Medical University (Army Medical University), Chongqing, China; ^4^Department of Neurosurgery, The First Affiliated Hospital of Chongqing Medical University, Chongqing, China; ^5^Department of Neurology, Xinqiao Hospital, Third Military Medical University (Army Medical University), Chongqing, China

**Keywords:** subarachnoid hemorrhage, white matter injury, oligodendrocyte, diffusion tensor imaging, therapeutic targets

## Abstract

Aneurysmal subarachnoid hemorrhage (SAH) is one of the special stroke subtypes with high mortality and mobility. Although the mortality of SAH has decreased by 50% over the past two decades due to advances in neurosurgery and management of neurocritical care, more than 70% of survivors suffer from varying degrees of neurological deficits and cognitive impairments, leaving a heavy burden on individuals, families, and the society. Recent studies have shown that white matter is vulnerable to SAH, and white matter injuries may be one of the causes of long-term neurological deficits caused by SAH. Attention has recently focused on the pivotal role of white matter injury in the pathophysiological processes after SAH, mainly related to mechanical damage caused by increased intracerebral pressure and the metabolic damage induced by blood degradation and hypoxia. In the present review, we sought to summarize the pathophysiology processes and mechanisms of white matter injury after SAH, with a view to providing new strategies for the prevention and treatment of long-term cognitive dysfunction after SAH.

## Introduction

Aneurysmal subarachnoid hemorrhage (SAH) is one of the special stroke subtypes with high mortality and mobility. Neurosurgical clipping or endovascular coiling is highly recommended for the early repair of ruptured aneurysms (1), focusing the medical management of SAH patients on early brain injury and delayed cerebral ischemia (2). However, more than 70% of survivors suffer from varying degrees of neurological deficits and cognitive impairments, leaving a heavy burden on individuals, families, and the society (3). Compared with cohorts with unruptured intracranial aneurysm, patients with aneurysmal SAH have higher mean diffusivity in white matter, leading to cognitive impartment 3 months after SAH onset (4). Apparently, the mammillothalamic tract is more vulnerable than the corticospinal tract in SAH patients with a good Glasgow Outcome Scale at 3 months after ictus (5), which demonstrates a correlation between early brain injury and long-term cognitive dysfunction after SAH.

White matter contains most of the volume of human brain and is made up of neural axons and myelin sheath. As early as 1989, the autopsy of six SAH cases had reported the remarkable hyperemia and edema in the deep frontal white matter, with microscopic axonal degeneration (6). Despite that enormous progresses have been made in the pathophysiology of early brain injury after SAH, the mechanisms of white matter injury are still a blur (7). Unlike intracerebral hemorrhage and traumatic brain injury, most SAH patients, especially those without obvious hematoma volume, do not usually fracture the nervous tract due to primary mechanical stress but suffer with remarkable secondary brain injury and neurological deficits. Mechanical pressure due to increased intracerebral pressure, glial response, and ischemia is considered as the pivotal mechanism of white matter injury after SAH but lacks high-quality clinical and basic research evidence (7).

In the present review, we sought to summarize the pathophysiology processes and mechanisms of white matter injury after SAH, with a view to providing new strategies for the prevention and treatment of long-term cognitive dysfunction after SAH.

## White Matter Injury After SAH

Previous clinical studies have not significantly improved neurological outcomes in patients with SAH. Recent studies have shown that there is a significant white matter injury after SAH, which plays an important role in the early brain injury secondary to SAH. The development of stroke imaging techniques suggests that the protection of white matter injury is very important for the recovery of neurological function and prognosis in patients with SAH. However, the relationship between white matter injury and SAH remains unclear. It has been reported that common cognitive dysfunction after SAH may be caused by white matter injury (8). Studies have shown that white matter injuries such as demyelination and axial rupture are reversible to a certain extent (9), while gray matter injuries such as neuronal apoptosis are difficult to recover. Therefore, the study of white matter injury after SAH may be of more importance than we currently know. Lee et al. found extensive white matter abnormalities in SAH patients through tract-based spatial statistical analysis, but not in retrolenticular parts of the internal capsule, right superior longitudinal fasciculus, or right superior corona radiata (10), providing important data support for the accurate diagnosis of the presence and severity of nerve injury in patients with subarachnoid hemorrhage. Clinical studies did find different types of white matter lesions in SAH patients. For example, the white matter lesions around the ventricle are mostly moon-shaped, thin-shaped, or cap-shaped, and these small spots or caps are asymptomatic and progress slowly (11); the white matter lesions in the deep brain are mostly patchy, macular, or fused large masses which progress rapidly and lead to long-term disease (11). In addition, autopsy observations of death cases with SAH showed white matter edema and demyelination (12). Brain tissue from a patient who died in the acute phase of SAH showed multiple subcortical white matter abnormalities in the brain, cerebellum, and brainstem (13). Distinct kinds of white matter injuries lead to cognitive dysfunction, memory loss, emotional apathy, movement disorders, and many other related clinical manifestations in SAH patients (11).

Retrospective quantitative MRI studies have also shown diffuse vasogenic edema and white matter injury after SAH (14). In recent years, diffusion tensor imaging (DTI) has been used to evaluate the neural tracts and structures in SAH patients (15). Jang et al. found 62.5% of SAH patients had at least one hemisphere of mammillothalamic tract abnormal 6 weeks after SAH onset (16). Some other SAH patients may have severe memory impairment and provoked confabulation in clear consciousness since SAH onset, with Papez circuit injury even 3 months later (17). In addition, Schweizer et al. also reported a reduction in hippocampal white matter integrity and long-term memory loss of SAH patients (18). In 2007, Liu et al. suggested that SAH may cause whole brain edema in the deep gray and white matter (19). Subsequently, Rejmer et al. reported that the mean white matter diffusion rate 2 weeks after onset in 49 patients with aneurysmal SAH was higher than that in 22 patients with unruptured aneurysms (4). Moreover, the gray matter/white matter ratio has been proved to be a good predictor for long-term cognitive function and quality of life in SAH patients (20). These studies suggest that it is essential to pay attention to white matter injury for the recovery of neurological function after SAH.

## Potential Pathophysiology Mediated Secondary White Matter Injury After SAH

Acute white matter injury in ICH and TBI is similar in principle and is caused by barotrauma and physical expansion of hematoma masses (21, 22). The difference is that barotrauma in TBI is caused by a blow to the outside of the head, whereas ICH is caused by the impact of a large amount of blood rushing out of the arteries in the brain cavity (21). In addition, neuroinflammation, oxidative stress, and excitatory toxicity induced by hematoma effect played major roles in the injury of secondary white matter (23). White matter, especially deep white matter, receives less collateral circulation than gray matter and is more sensitive to ischemia (24). Thus, ischemic stroke damages white matter rapidly and profoundly. In addition, oliodendrocytes are highly susceptible to cerebral ischemia-induced oxidative stress (25), excitotoxicity (26), and neuroinflammation, leading to oliodendrocyte apoptosis and consequent white matter damage.

**Figure 1:**
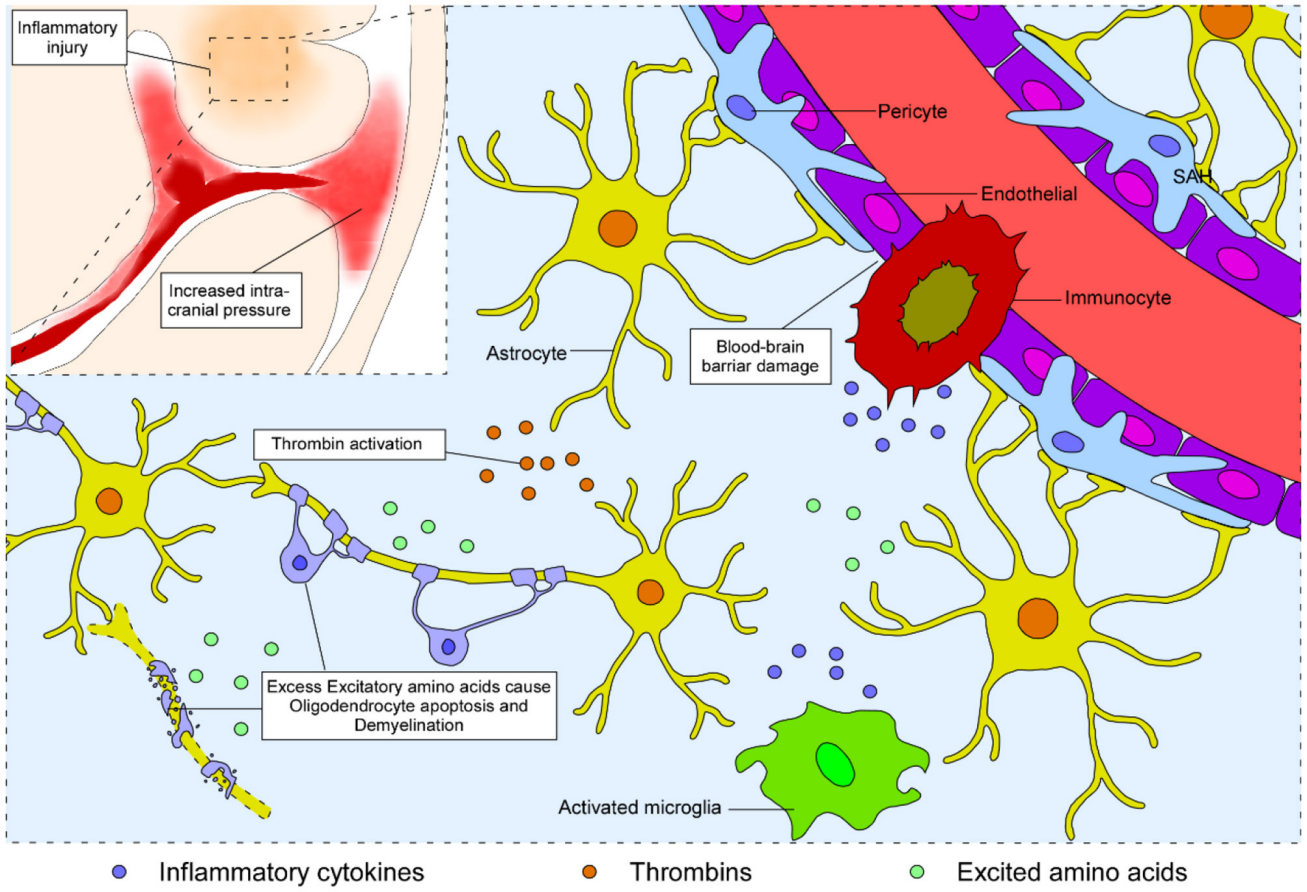
Schematic representation of the process of white matter injury in the brain after SAH. Blood from the ruptured vessel enters the subarachnoid space and increases the intracranial pressure. Subsequently, biochemical factors, including thrombin, excitatory amino acids, and inflammatory cytokines, lead to disruption of the blood–brain barrier, neural apoptosis, and demyelination, eventually causing white matter injury after SAH.

Since most blood spread into the subarachnoid space without direct nervous tract disruption, white matter injury after SAH is initially considered to be the consequence of blood–brain barrier disruption (27, 28) and neurotoxicity of blood disintegration (29). Physical factors such as biological stress, mainly caused by elevated intracranial pressure, attack the whole brain in the acute phase, while biochemical factors including thrombin, excitatory amino acids, and inflammatory cytokines lead to subsequent white matter injury after SAH (Figure 1).

### Elevated Intracranial Pressure

Changes occurring within 1 min after aneurysmal SAH cause early brain injury, and the degree of early brain injury affects the final prognosis of patients (30). There is evidence that acute intracranial hypertension after SAH is often accompanied by sudden loss of consciousness and worse prognosis (31). Therefore, control of intracranial pressure in the acute phase of SAH is helpful to improve patient prognosis. Blood breaks into the enclosed cranial cavity after SAH, causing a sharp increase in intracranial pressure (ICP). Cerebral white matter midline displacement directly caused by uneven pressure causes the brain tissue to bulge into the tentorium cerebellum and foramen magnum, inducing the herniation of the supraoptic temporal lobe or cerebellar tonsillar hernia, and then squeezes the brain stem and respiratory center, resulting in acute crisis, loss of consciousness, respiratory depression, and cardiac arrest (32). Therefore, almost one-third of patients suffering from SAH consequently die of cerebral hernia due to high intracranial pressure. The indirect effects of high intracranial pressure on white matter after SAH seem clear as well. First, elevated intracranial pressure leads to neurovascular coupling disorders, reducing the focal cerebral blood flow and blood supply to the white matter (33). Second, high intracranial pressure disrupts the blood–brain barrier (BBB) at a very early phase after SAH. Excessive water (H[[sb]]2[[/s]]O) and Na+ diffuse into the brain parenchyma *via* the impaired BBB, resulting in vasogenic white matter edema. Edema also aggravates intracranial pressure again after SAH (34). Third, increased intracranial pressure causes the extrusion of H[[sb]]2[[/s]]O into the extracellular mesenchyme along the white matter fiber bundles, enhancing interstitial cerebral edema following SAH (33, 35). Since cerebrospinal fluid penetrates the ventricle wall and infiltrates the white matter surrounding the ventricle, it increases hydrostatic pressure and results in myelin disintegrating and disappearing rapidly (34). Finally, increased intracranial pressure suppresses the venous and lymphatic systems, leading to obstruction of venous and lymphatic influx, further increasing cerebral edema and intracranial pressure (36).

It is of great importance to develop intervention strategies that target pathogens to improve ICP-induced white matter injury after SAH. The primary cause is the occupancy effect of excess blood in the subarachnoid space. Hence, early intracranial hematoma evacuation and cerebrospinal fluid drainage can alleviate white matter damage after SAH. Another cause of increased ICP after SAH is hydrocephalus. Both traffic hydrocephalus and non-traffic hydrocephalus aggravate the increase in ICP (34) and further compress the central aqueducts, impeding the absorption and circulation of cerebrospinal fluid (37). Therefore, a lumbar puncture and drainage procedure or application of diuretics is urgently needed to reduce white matter injury after SAH. As mentioned above, mitigation of brain edema is beneficial to reduce the damage of intracranial pressure on the white matter. However, at present, hormone drugs based on improvement of the BBB are not recommended because of their disadvantages, such as poor specificity and side effects (38).

### Thrombin

Thrombin plays inclusive roles in the human brain, including coagulation cascades and non-clotting processes. Under physiological conditions, thrombin exists in the form of prothrombin participating in the endogenous and exogenous coagulation cascade (39). Thrombin is also involved in non-clotting processes, such as maintenance of the blood–brain barrier, aggregation and activation of platelets, formation of cerebral edema, inflammatory cell infiltration, physiological proliferation, and repair of brain tissue (40). Cerebrovascular spasm is highly correlated with the prognosis of SAH patients (41), and thrombin plays an important role in it (42). The stimulative effect of thrombin on vasospasm after SAH is irreversible (43). Therefore, how to reduce the enhancing effect of thrombin on vasospasm after SAH so as to reduce the subsequent toxic effect and improve the prognosis of SAH patients remains to be further studied.

Excessive activation of thrombin during pathological scenarios shows neurotoxic effects. First, thrombin activates its membrane receptors and accumulates intracellular calcium (Ca^2+^), which activates the calcium-dependent apoptosis signaling pathway, killing the neurons and glial cells (44). Second, thrombin activates matrix metalloproteinases (MMPs), which may degrade various extracellular matrix proteins (such as tight junction proteins), increasing blood–brain barrier permeability and aggravating posterior cerebral edema after SAH (45). Third, activation of microglia in the brain and peripheral blood immune cells, which enter the central nervous system through an impaired blood–brain barrier, may cause serious neuroinflammatory injury (46). Fourth, studies regarding ischemia–reperfusion injury in mouse models have reported that astrocytes originally activated by thrombin may aid in the production of MMP-2 and reduce myelin cells (47). The MMP-2 inhibitor reduced the proliferation of astrocytes and the damage of myelin cells but failed to reduce the damage to oligodendrocytes caused by thrombin (47), which suggests that the damage of thrombin to oligodendrocytes may occur through apoptotic or inflammatory pathways rather than through the MMP pathway.

Although thrombin receptor inhibitors should work against white matter injury after SAH, very few antagonists of thrombin receptors have been applied to SAH patients because of the risk of secondary hemorrhage (48). Furthermore, most small molecular inhibitors of thrombin have been found to be effective in animal experiments, yet failed in clinical trials (49). Recent research in our laboratory found that the direct use of antagonist peptides of the thrombin receptor in a mouse model of SAH promoted remyelination and neurological recovery (50). However, in consideration of the substantial differences among species, there is still a long way to go to achieve a clinical transformation.

### Blood–Brain Barrier Damage

Approximately 10% of SAH patients suffer from severe edema in many cerebral regions, including the white matter, cortical cortex, and corpus callosum (51). Vasogenic edema caused by disruption of the blood–brain barrier is an independent risk factor for death and disability in SAH patients (52). The blood–brain barrier maintains the homeostasis of the internal environment of the human brain with its particular structure: a basal membrane, pericytes, an endothelium, and astrocytic endfeet (53). An ultrastructural observation showed changes in the blood–brain barrier after SAH, including a decrease in the number of pericytes, a lack of connection between the pericytes and the endothelium, disintegration of the astrocytic endfeet, destruction of the tight junctions of the endothelium, degradation of the basal membrane, vacuole-like changes in the endothelial cells, and plasma leakage (54). Furthermore, some phenomena, such as myelin edema, axonal energizing and information transmission disorders, and disintegration of white matter fibers, have also been observed after SAH (55). Interestingly, the MMPs that cause destruction of the endothelial tight junctions and disruption of the BBB have shown promotion of remyelination in the peripheral nervous system (56, 57), suggesting that the potential mechanism of correlation between BBB damage and white matter injury remains elusive in SAH scenarios.

Two mechanisms have been involved in BBB maintenance: tight junction and transcytosis (58). The MMPs, as tissue scissors, regulate the integrity of tight junctions, while docosahexaenoic acid (DHA) suppresses endothelial transcytosis (58). Our previous works found that both mechanisms were apparent in the regulation of the BBB in white matter (where it is adjacent to the blood clot directly) after SAH (58). Moreover, capillary pericytes are involved in the two mechanisms by secreting matrix metalloproteinase-9 (MMP-9) and DHA, revealing an interesting role of pericytes in white matter injury after SAH.

### Excitatory Amino Acids

Glutamate is the main excitatory amino acid (EAA) of the human brain. Glutamatergic neurons participate in mediating motor conduction in the spinal cord, red nucleus giant cells, and Deiters' nucleus giant cells (59). The concentration of glutamate is abundant (up to 10 μmol/g brain tissue) in the human central nervous system (60). Its receptors (glutamate receptor, GluR) include the metabotropic (mGluR) and ionic (iGluR) receptors (60). The former, mGluR, is a G protein-coupled receptor that activates protein modification and regulates the process of learning, memory, anxiety, and pain transmission. The latter, iGluR, is a ligand-gated ion channel that is further subdivided into three subtypes: the N-methyl-D-aspartate receptor (NMDAR), aminohydroxymethyl oxazole propionate receptor (AMPAR), and kainate receptor (KAR). Studies have found that both mGluR and iGluR are widely distributed in white matter (61). In pathological scenarios, excess glutamate is released from damaged glutamatergic neurons, causing demyelination, axonal injury, and glial cell death (62). Furthermore, the blocking of reuptake and physiological elimination of glutamate also lead to its excessive accumulation (62). This reuptake failure is mainly because of the functional inhibition of the presynaptic membrane of neurons or the transporter of glutamic acids (GluTs, which consume ATP) on the glial cell membrane (62). In the ischemic and hypoxic environment caused by stroke, ATP supply is disturbed, and glutamate reuptake is weakened (62). However, the antiport of the sodium-dependent glutamate transporter further aggravates the accumulation of glutamate and causes cell excitotoxicity (62). Excessive glutamate leads to intracellular calcium overload, oxidative stress, and endoplasmic reticulum stress, resulting in oligodendrocyte apoptosis, and demyelination (62).

Excess glutamate is found in cerebrospinal fluid and brain tissues of SAH patients. A correlation study analyzed the level of excitatory amino acids in the intercellular substance of SAH patients using microdialysis and found that the elevation of aspartic acid and glutamate levels was negatively correlated with the prognosis of SAH patients (63). Another animal experiment showed that the expression of mGluR and the glutamate transporter decreased significantly in the early stage of SAH (64). In their report, upregulation of the glutamate receptor and its transporter by using magnesium sulfate or nimodipine significantly improved neurological functions (64). These studies suggest that glutamate levels in the brain may be an important indicator of white matter injury after SAH.

### Inflammatory Injury

Neuroinflammation has been shown to be an important pathogenic factor for white matter injury after SAH (65). A case comparison study by Leviton and colleagues classified neonatal intracerebral hemorrhage into three types according to whether there was white matter injury: cerebral hemorrhage with white matter injury (68 cases), cerebral hemorrhage without white matter injury (123 cases), and no cerebral hemorrhage or white matter injury (1,677 cases). This study found that the inflammatory response was more significant and lasting in patients who had white matter injury (66). After SAH, the majority of inflammation was induced by the polarization of microglia from the resting state (M0 type) to the immune damage state (M1 type) or neuroprotection state (M2 type) (67). Therefore, it was a promising direction of inflammatory therapy by converting the polarization of microglia after SAH (68). Peng et al. recently reported that low-density lipoprotein receptor-related protein-1 activation could modulate M2 microglial polarization and attenuate white matter injury after SAH (69), and apolipoprotein E and its mimetic peptide COG1410 could reduce M1 microglia activation for the protection of white matter injury after SAH (70). In addition, peripheral lymphocytes, such as T lymphocytes and macrophages, penetrated into the brain through the damaged blood–brain barrier after SAH (71). These immune cells either killed or phagocytosed neurons and glia, causing white matter inflammatory injury (71). Microglia secrete proinflammatory factors such as TNFα, NIL1, and IL13, leading to oligodendrocyte and periventricular white matter injury in ischemic circumstances (72). Activation of microglia also transformed inactive MMPs into active MMP-3 and MMP-9, thereby destroying the BBB and degrading myelin (73). In addition, intervention of inflammatory injury after SAH should be undertaken as early as possible since inflammation can interfere with the videographic diagnosis of SAH (74).

## Repair Factors of White Matter After SAH

### Oligodendrocyte Precursor Cells

Oligodendrocytes are responsible for the myelination of axons. During the development of the brain, oligodendrocytes usually originate from the neuroepithelial area where neural stem cells differentiate into oligodendrocyte precursor cells (OPCs) before developing into early oligodendrocytes (75). Recent studies have shown that OPCs develop in stages and form potentially diverse populations (76). OPCs from different sources have different susceptibilities and transcriptional profiles (76). If some OPCs are more sensitive than others, there may be a promising therapeutic strategy to target the vulnerable OPC subpopulation. OPCs, which still exist in the adult brain, are crucial for myelin maintenance and can be recruited for remyelination in the case of myelin damage (77). Moreover, in adult mice, OPCs account for ~8–9% of the white matter cell population and 2–3% of the gray matter cell population, suggesting that OPCs are the most important cells mobilized in remyelination after brain injury (77).

After proliferation, most OPCs are integrated into neural circuits, and excessive ones are removed by microglia (78). In addition, it has been shown that myelination is a dynamic and plastic process (79). For example, studies in animals and humans have shown that neural activity facilitates the differentiation and myelination of OPCs during exercise and learning (79). OPCs have a gross cone-like structure and can reach damaged areas under the guidance of multiple chemokines (80). For example, the concentration gradients of bone morphogenetic protein (BMP), Sonic hedgehog (Shh), and Wnt protein determine the direction of migration of OPCs (80). Other factors, such as growth factors, extracellular matrix proteins, axon-inducing molecules, and neural activity, can also influence the migration of OPCs (77). In addition, it has been shown that the migration of OPCs is also stimulated by extracellular matrix components such as laminin, fibronectin, vitronectin, anosmin-1, and tenascin-C (81). Interestingly, glutamate can promote the migration of OPCs by stimulating the expression of polysialic acid–neuronal cell adhesion molecules and activating the Tiam1/Rac1/ERK signaling transduction pathway (82). A recent study by Tsai and colleagues showed that correct cerebral vascularization was essential for the migration of OPCs (83). More specifically, OPCs migrated by “crawling” along the blood vessels and could also “jump” from one blood vessel to another (83). This behavior of OPCs may aim to ensure an adequate oxygen supply during the myelination process, which requires a high oxygen consumption. Xu and colleagues recently reported that perioxisomal dysfunction exacerbated white matter injury after SAH, at least partly through thioredoxin-interacting protein and glycerone phosphate acyl transferase signals (84).

**Figure 2:**
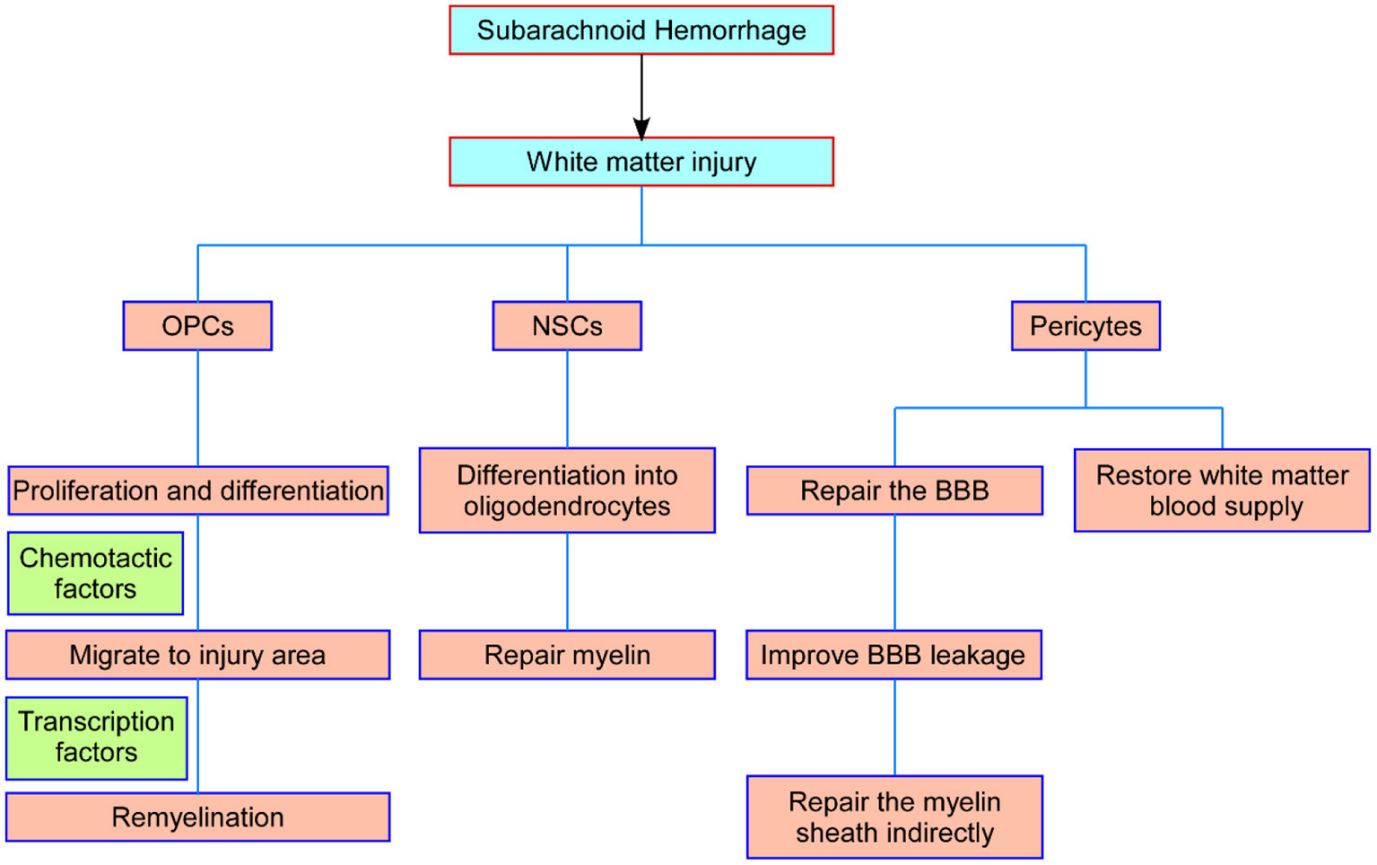
Schematic representation of the main processes of NSCs, OPCs, and pericytes in white matter repair after SAH. OPCs proliferate and migrate to the injured foci and differentiate into oligodendrocytes to repair damaged myelin. NSCs differentiate into white matter neurons and oligodendrocytes to repair axons and myelin. Pericytes restore the blood supply to the white matter by modulating capillary constriction; moreover, pericytes enhance BBB integrity and alleviate white matter injury after SAH. NSCs, neural stem cells; OPCs, oligodendrocyte precursor cells; SAH, subarachnoid hemorrhage; BBB, blood–brain barrier.

OPCs will not stop proliferating after reaching their destination until the number of OPCs reaches homeostasis (75). OPCs are in a state of moderate proliferation and differentiation inhibition. PDGF signaling is the main inducer of OPC proliferation, while Notch and Wnt signaling and downstream transcription factors are inhibitors of OPC differentiation (85, 86). The inhibition of OPC differentiation is relieved after white matter injury, and OPCs differentiate into immature oligodendrocytes and eventually form myelin sheaths under the promotion of transcription factors such as myelin regulators (Myrf) (87). Mature oligodendrocytes ultimately achieve myelin assembly of axons by expressing a large number of myelin genes after contact with neuronal axons, including myelin-associated glycoprotein (MAG), myelin oligodendrocyte glycoprotein (MOG), myelin basic protein (MBP), and myelin protein lipoprotein (PLP) (88). Therefore, the proliferation, migration, differentiation, and maturation of OPCs, as well as the internal regulatory mechanism, could provide new strategies for plastic myelin regeneration after SAH (Figure 2).

### Neural Stem Cells

Neural stem cells (NSCs) are located in the stem pool (subventricular zone and hippocampus) of the brain and are considered to be the center of cerebral regeneration (89). Surprisingly, NSCs are the source of myelin repair by differentiating into oligodendrocytes (90). Stem cell therapy has been confirmed in rescue organs and tissues in a variety of animal models (90). However, most of these therapies are still experimental, and only small-scale and experimental stem cell therapy trials have been conducted in clinical practice (90). Furthermore, some issues that are not negligible, including directional differentiation, side effects, and risk of immune rejection, have not been resolved at present.

Compared with other described therapeutic cells, including embryonic stem cells, mesenchymal stem cells, umbilical cord stem cells, and induced pluripotent stem cells, human NSCs have shown the advantage of stable proliferation, mainly differentiating into neurons and oligodendrocytes. Studies have shown that nasal administration of mesenchymal stem cells (MSCs) after 6 days of SAH in rats significantly reduced brain injury and neuroinflammation and improved neurofunctional outcomes after 21 days of SAH (91). The first case of venous stem cell treatment of a high-grade aneurysmal SAH patient indicated that the patient recovered rapidly and well after intravenous infusion of bone marrow-derived allogeneic MSCs on day 3 after hemorrhage and achieved a modified Rankin Scale score of 3 at 6 months (92). The immunological rejection of neural stem cell transplantation may be improved by gene editing in the future, and we may quickly obtain a large number of neurons by using iPSC technology, reducing costs and shortening the transplantation time (93). These clinical trials suggest that neural stem cells have great clinical application value and should be used as one of the treatment options for white matter injury.

In recent years, some achievements have been made in basic and clinical studies of stem cell therapy, but there are still some limitations. The safety and reliability of stem cell therapy, especially the safety of long-term treatment, need to be verified by more experiments. Stem cell type, delivery location and route, and optimal time of intervention of stem cell therapy need to be further explored. There are great challenges and still has a long way to go from animal experiments to clinical applications, but we believe that these findings provide a certain basis and guidance for stem cell therapy in the treatment of white matter injury after SAH (Figure 2).

### Pericytes

The pericytes surrounding capillaries are essential for maintaining the structure and function of the blood–brain barrier. Pericytes have been found to be involved in white matter injury in cerebral arterial disease with subcortical infarcts and leukoencephalopathy, cerebral ischemia, and primary brain calcification (94). Deletion of beta-type platelet-derived growth factor receptor (PDGFRb) causes pericyte loss and affects white matter functions in two ways: one is that the loss or dysfunction of pericytes leads to blood–brain barrier leakage, causing further toxic injury and white matter edema (95); the other is that pericyte contraction causes microcirculatory disturbance, blocking the white matter blood supply and causing white matter ischemia and hypoxia damage (96). Under pathological conditions, pericytes can be transformed into α-smooth muscle actin (a-SMA)-positive phenotype, showing the regulation of contractile function and leading to neurological impairment (97). Our previous studies have shown that regulating eNOS/NO signal can inhibit the conversion of pericytes to a-SMA-positive phenotype, thereby increasing the diameter of capillaries and regulating the neurological dysfunction caused by microcirculation disturbance after SAH (98). Moreover, increased expression and secretion of MMP-9 in pericytes after SAH degrades endothelial tight junction protein and basement membrane (99), while cyclosporine A (CsA) can improve nerve injury caused by vasogenic edema by inhibiting this process (58). The activation of oxidative stress induced by SAH leads to increased apoptosis of pericytes, and edaravone can inhibit this change and improve the early brain injury after SAH (100). In addition, pericytes also show stem cell properties and differentiate into neurons under certain conditions and further repair nerve damage (101). Therefore, pericytes have great potential in the treatment and intervention of white matter injury (Figure 2).

## Potential Therapeutic Strategies Targeting White Matter Injury After SAH

Currently, clinical trials on SAH mainly focus on alleviating vascular spasm, and there are few studies related to white matter injury. The study on the neuroprotective effect of ketamine infusion after aneurysmal SAH is in phase 3 clinical trial now, which will provide an effective treatment for the protection of neurocognitive function in patients with SAH after completion (ClinicalTrials.gov identifier: NCT02636218). Moreover, the effect of Xenon treatment on brain injury in the acute phase after aneurysmal SAH is in the initial phase of a phase 2 clinical trial and is expected to be a new approach for treating white matter injury after SAH (ClinicalTrials.gov identifier: NCT04696523).

In addition, there are a variety of drugs that promote remyelination or impedance demyelination in preclinical and clinical application stages. For example, bazedoxifene (BZA) is one of the third-generation selective estrogen receptor modulators that has been shown in preclinical studies to promote myelin regeneration (102) and is currently undergoing phase II clinical trials in MS patients (ClinicalTrials.gov identifier: NCT04002934). In an animal model of cuprizone-induced demyelination, testosterone promotes the proliferation and differentiation of OPCs into mature oligodendrocytes through targeting neuroandrogen receptors (103). Due to the potential side effects and risk of overdose of hormone therapy, the phase II clinical study of testosterone is currently being conducted only in patients with testosterone deficiency, with the primary objective of determining the efficacy of testosterone in MS (ClinicalTrials.gov identifier: NCT03910738). Experiments have shown that metformin can promote the expansion, migration, and differentiation of endogenous neural progenitor cells in injured rodent brains, so as to carry out self-repair and functional recovery (104). The research on the effect of metformin on endogenous neural progenitor cells in patients with multiple sclerosis is undergoing phase II clinical trial (ClinicalTrials.gov identifier: NCT04121468). Canavan disease is a congenital white matter disorder characterized by severe motor abnormalities and low myelination, and a phase II clinical trial of RAAV-Oligo001-ASPA in the treatment of Canavan disease has just initiated (ClinicalTrials.gov identifier: NCT04833907). RAAV-Olig001-ASPA is the first gene therapy targeting oligodendrocytes, which are critical for myelination and brain development (105). A phase II clinical trial of nanocrystalline gold for multiple sclerosis has initiated (ClinicalTrials.gov identifier: NCT03536559). If these clinical trials are successful, the high specificity of gene therapy and strong targeting of nanomaterials will provide new therapeutic strategies for remyelination after SAH. Besides, clomastine, solinacine, and benzotropine have been tested in preclinical studies and approved for clinical use. Other promising drugs, such as VX15/2503, BIIB033, and GSK239512, promoted the differentiation of oligodendrocyte precursor cells by inhibiting Wnt, Notch, and other signaling pathways *in vitro*/animal experiments, thus promoting myelin regeneration. However, no relevant clinical studies have been conducted so far. Several drugs and their targets are summarized in Table 1.

**Table 1:** Advances in drug research for myelin repair.

**Medicines**	**Targets**	**Mechanism**	**Current state**	**Correlational research**
Fingolimod	S1P1 receptor	Promotion of the maturity of oligodendrocytes	FDA approved for RRMS	(106)
Benzatropine	Notch signal; muscarinic receptor	Reduction in cholinergic demyelination; promotion of the differentiation of OPC	Approved for Parkinson's, dystonia, and EAE	(107)
Quetiapine hemifumarate	D2, 5-HT2A antagonist, H1, α1, and 5-HT1A receptor	Promotion of the proliferation and maturation of oligodendrocytes; increased antioxidative stress	Approved for schizophrenia, bipolar disorder; MS patient phase I clinical trial (ClinicalTrials.gov: NCT02087631)	(108)
Simvastatin	3-Hydroxy-3-methylglutaryl-CoA (HMG-CoA) reductase inhibitor	Reduction in brain atrophy SPMS; reduction in recurrent frequency or lesion load	Approved for hypercholesterolemia; phase III clinical trials of SPMS (NCT00647348)	(109)
Clobetasol	Corticosteroid receptors	Promotion of the differentiation of OPC cells	Local antimicrobials; no clinical studies have been conducted	(110)
Indomethacin	Nonsteroidal anti-inflammatory drugs; drug (NSAID) inhibits cyclic oxidase	Increase the phosphorylation of β-catenin and induce its degradation; promotion of differentiation of OPC cells	Approved as an OTC pain reliever. No clinical studies have been conducted	(111)
BIIB033	LINO-1; RhoA signal	Enhancement of oligodendrocyte maturation, myelin formation, and reduction in severity of EAE	MS phase II clinical trial (NCT01864148); phase I of optic neuritis (NCT01721161)	(112)
Clemastine	Antihistamine/anticholinergic compounds; blocks histamine H1 receptor	Enhancement of OPC cell differentiation	RRMS patient II clinical trial (NCT02040298)	(113)
Solifenacin	Blocks CHRM3, an M3R muscarine acetylcholine receptor	Enhancement of OPC cell differentiation	FDA approval for contractive bladder contraction; no clinical studies have been conducted	(114)
BQ788	Endothelin (ET) receptor antagonist	Blocking of astrocytes and oligodendrocyte demyelination	*In vitro*/animal experimental evidence, no clinical application or related test	(115)
IRX4204	Retinoic acid receptor g (RXR-g)	Enhancement of oligodendrocyte differentiation	Clinical trials of MS patients are in the planning stage	(116)
VX15/2503	SEMA4D/plexinB1 signal	Promotion of OPC differentiation; repair of the BBB	MS patient phase I clinical trial (NCT01764737)	(117)
rHigM22	Hypoprotein/fibronectin receptor	Reduction in glial cell apoptosis; promotion of the regeneration of myelin	MS patient phase I clinical trial (NCT01803867)	(118)
GSK239512	Histamine H3 receptor agonist	Promotion of OPC differentiation	Patients with MS were given an additional therapy trial for the glatiramer acetate or interferon b-1a, which was completed in phase II (NCT01772199)	(119)

## Perspective and Conclusion

Previous studies have focused on early brain injury (EBI) and delayed cerebral ischemia (DCI) in patients with SAH. Most of these studies focus on the cortical gray matter neurovascular units or white matter neurons and ignore the myelin sheath regeneration and the structure and function of white matter fiber tracts. While <20% of the volume of white matter is present in commonly used laboratory rodents, white matter makes up more than 50% of the human brain. From the perspective of clinical treatment, even if damage of gray matter can be restored, the treatment of patients is difficult to achieve ideal results without repair of white matter fiber bundle and myelin sheath. These studies suggest that it is important to pay attention to white matter injury for neurological rehabilitation after subarachnoid hemorrhage.

The therapeutic strategies for white matter injury are mainly limited to maintaining the balance of damage and reconstruction of white matter components after SAH ictus. The present review discusses the pathogenic factors and repair mechanisms of white matter injury after SAH, as well as the anti-inflammatory mechanisms to mitigate existing damage by reducing blood–brain barrier destruction, decreasing intracranial pressure, blocking thrombin-activating molecular cascades, and other means. On the other hand, regeneration of the myelin sheath and the repair of neurovascular can be promoted through the repair and functional stability of OPC cells, neural stem cells, and pericytes. The combination of these two strategies can reduce white matter injury after SAH and accelerate the recovery of neurological function. Potential drugs have been approved for clinical use and are expected to be used in the treatment of white matter injury after SAH. Given these exciting works, especially in preclinical studies, new breakthroughs in the treatment of neurological deficits caused by white matter injury in SAH patients may be possible in the near future.

## Author Contributions

XR, LG, JZ, QL, SZ, YC, and ZL draft the manuscript and figures. HF, ZL, and YC proof read and revise the manuscript. YC and ZL give the final prove for this submission. All authors contributed to the article and approved the submitted version.

## Conflict of Interest

The authors declare that the research was conducted in the absence of any commercial or financial relationships that could be construed as a potential conflict of interest.
